# Effects of two kinds of fishery drugs on the expressions of GAD and GABA-T mRNA in crucian carp (*Carassius auratus gibelio*)

**DOI:** 10.1007/s10695-020-00847-z

**Published:** 2020-07-15

**Authors:** Fan Zhang, Kun Hu, Jianzhen Huang, Zhi Tan, Jiming Ruan

**Affiliations:** 1 Department of Aquaculture, College of Animal Science and Technology, Jiangxi Agricultural University, Nanchang, 330045 China; 2 National Center for Aquatic Pathogen Collection, College of Fisheries and Life Science, Shanghai Ocean University, Shanghai, 201306 China

**Keywords:** *Gamma-*aminobutyric acid, Difloxacin, Avermectin, Glutamate decarboxylase, GABA-transaminase

## Abstract

The objective of this study was to investigate the effects of difloxacin (DIF) and avermectin (AVM) on glutamate decarboxylase (GAD) and GABA-transaminase (GABA-T) in different tissues of crucian carp (*Carassius auratus gibelio*). After the treatments of DIF and AVM, the mRNA expressions of GAD and GABA-T in different tissues were detected by quantitative real-time PCR (qPCR). The results showed that the mRNA expressions of GAD_65_, GAD_67_, and GABA-T in the telencephalon (Tel), mesencephalon (Mes), cerebella (Cer), and medulla oblongata (Med) were downregulated significantly with the safe dose (SD, 20 mg/kg) of DIF (*P* < 0.05 or *P* < 0.01). While the expressions of GAD_65_ and GAD_67_ in the kidney at 12 h had strikingly upregulated to 13.81 ± 1.06** and 150.67 ± 12.85** times. Treated with the lethal dose of 50% (LD_50_, 2840 mg/kg b. W.) of DIF, the mRNA expressions of GAD_65_, GAD_67_, and GABA-T in all tissues were increased significantly (*P* < 0.01). The results of AVM group showed that the mRNA expressions of GAD_65_, GAD_67_, and GABA-T both in the central and peripheral tissues were all remarkably downregulated at the safe concentration (SC, 0.0039 mg/L) and the lethal concentration of 50% (LC_50_, 0.039 mg/L), except for the mRNA inhibitions of GAD_65_, GAD_67_, and GABA-T in the muscle at 2 h which sharply downregulated to 0.20 ± 0.02^ΔΔ^ × 10^−2^, 0.57 ± 0.06^ΔΔ^ × 10^−1^ and 0.44 ± 0.02^ΔΔ^ × 10^−1^, respectively (*P* < 0.01).

## Introduction

GABA, an important inhibitory neurotransmitter in many organisms, along with glutamate (Glu), is involved in the neuromodulation of most synaptic activity. GABA arises via decarboxylation of L-glutamate by glutamate decarboxylase (GAD) (Chung et al. 1992) and is metabolized subsequently via GABA-transferase (GABA-T) to succinic semialdehyde, which is then oxidized to succinate (Wood et al. 1978). This process would directly affect the accumulations of GABA in organisms. The changes of Glu and GABA in nerve endings would result in rearrangements of the nervous system that increases neural activity (Nasreen et al. 2012). The production and metabolism of GABA can be predicted by observing changes in the expression of enzymes present in nerve endings.

However, many factors affect how well GABA works, which include heavy metal (Struzyńska and Sulkowski 2004), antibiotics (Matsuo et al. 1998), insecticide (Sánchez-Borzone et al. 2017), and other biological toxins (Kudryavtsev et al. 2015). The imbalances of excitatory or inhibitory neurotransmitters caused by drugs can lead to nerve abnormalities, causing organisms to exhibit symptoms of nerve poisoning.

Fluoroquinolones (FQs) were widely used in aquaculture due to its good bactericidal effect in China. Its family includes *difloxacin* (DIF), *ofloxacin*, *pefloxacin*, *enoxacin*, *norfloxacin*, etc. However, the side effects of FQs have been widely reported in recent years, such as its muscular toxicity (Demetrious 2018), renal toxicity (Owens and Ambrose 2005), and neurotoxicity (Xiao et al. 2018). Many reports suggested FQs could have caused severe neurotoxic reactions, which lead to hallucinations, depression, and other neurological diseases (Barrett and Login 2009; Grill and Maganti 2011; Guiol et al. 1993). It had been reported that *norfloxacin* (Xi et al. 2019) and DIF (Ruan et al. 2014a) caused non-target biological neurotoxicity. Albino male mice treated with *ciprofloxacin* were found the levels of Glu and GABA significantly reduced (Arafa et al. 2015). While as a broad-spectrum insecticide, avermectin (AVM) was also widely used for parasites killing in aquaculture. The problems of non-target biological poisoning caused by AVM were becoming more and more serious, including in birds (de Faria et al. 2018a, b), fish (Novelli et al. 2016), batrachians (Vasconcelos et al. 2016), and mammals (Nasr et al. 2016). Experiments on *Danio rerio* (Weichert et al. 2017), mice (Da et al. 2018), and pigeon (Li et al. 2013) had been found that AVM could cause neurotoxicity. On specific physiological and biochemical indicators, AVM exposure enhanced the contents of GABA, glycine, Glu, and aspartic acid in the cerebrum, cerebellum, and optic lobe of American king pigeons significantly (Chen et al. 2014). Crucian carp (*Carassius auratus gibelio*) is one of the tremendous economic value fish which widely cultivated in China. Therefore, this paper intends to study the possible variations of GABA’s synthetase and metabolic enzyme in crucian carp after treated with DIF and AVM.

## Materials and methods

### Experimental animals and fishery drugs

Crucian carp were bought from a farm in Nantong City, Jiangsu Province, east of China as experimental fish, which body weights were 50.04 ± 3.12 g, and fed for 2 weeks before the beginning of the experiments. Plenty of oxygen was pumped into the water during the whole experiment. Temperature and pH values were maintained in the right range of crucian carp*.* All the fish were fed twice per day.

The SD and the lethal dose of 50% at 96 h (96 h LD_50_) of DIF were 20 mg/kg b. W. and 2840 mg/kg b. W. which referred from the previous study (Ruan et al. 2014a). According to the body weights of the experimental fish, DIF was singly orally administered into the foregut of the experimental fish.

The SC and 96 h LC_50_ of AVM were 0.0039 mg/L and 0.039 mg/L by the method of single bath administration (Ruan et al. 2013).

### The sample collections

The experimental fish were randomly divided into three groups at the corresponding dose/concentration, 60 fish per group. Samples of Tel, Mes, Cer, Med, liver, kidney, and muscle were collected and stored at − 80 °C for the mRNA extractions. All fish were handled following the “Regulation on Animal Experimentation.”

### Total RNA extraction, reverse transcription, RT-PCR analysis, and qPCR analysis

The procedures of total RNA extraction, RT-PCR, and qPCR analysis were referred from the previous article (Ruan et al. 2014b). The primers pairs were shown in the Table 1.

**Table 1:** Information of primers of the paper

Genes	Primer sequence(5′-3′)	GenBank ID	Size (bp)	Temp. (°C)
*β*-Actin	Forward TACGTTGCCATCCAGGCTGTG Reverse CATGGGGCAGGGCGTAACC	M24113.1	124	55–60
GAD_65_	Forward TTCTCTGTCGCTGCTCTGAT Reverse CTCTCGGCTGTAGACCCAT	AF149832.1	246	57.4
GAD_67_	Forward GTTTTCTGATATCAAGCGTCTCAC Reverse TGGCAGGTTGTCGTAAATTAG	AF149833.1	209	56.1
GABA-T	Forward GCTGCCTGGCCACAACACA Reverse TCCCTCACAAACTCCTCCAGA	DQ287923.1	115	57.5

### Data processing

The comparative threshold method (2^-ΔΔCT^) was employed to calculate the relative expression of the genes. The data were expressed as mean ± standard deviation (SD) and SPSS 17.0 (Chicago, IL, USA) was used for one-way ANOVA, where *P* < 0.05 and *P* < 0.01, respectively, indicated significant and extremely significant difference.

## Results and analysis

### Analysis of genes expressions in the brain at the SD of DIF

**Fig. 1:**
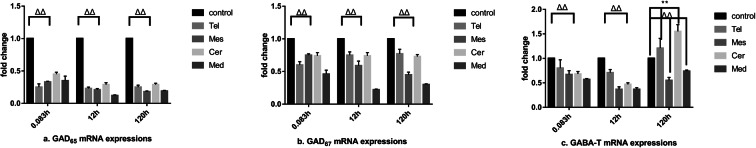
Effects in brain GAD and GABA-T mRNA expressions at the SD of DIF

Note: 0.083 h, 12 h, and 120 h were the three-time points of DIF at the SD (20 mg/kg b. W.) (Ruan et al. 2014a). “^Δ^” and “^ΔΔ^” mean significant (*P* < 0.05) or extremely significant (*P* < 0.01) downregulation, while “*” and “**” mean significant (*P* < 0.05) or extremely significant (*P* < 0.01) upregulation. The same as followed.

From Fig. 1 a and b, it could be found that GAD_65_ and GAD_67_ expressions were significantly suppressed (*P* < 0.01) in the crucian carp’s brain by SD of DIF (20 mg/kg b. W.) after treatment at 0.083, 2, and 120 h. The volumes and the time-points which GAD_65_ expressions remarkably inhibited were 0.25 ± 0.05^ΔΔ^ at 0.083 h in Tel, 0.12 ± 0.01^ΔΔ^ at 12 h in Med, and 0.18 ± 0.01^ΔΔ^ at 120 h in Mes, respectively (Fig. 1a). GAD_67_ levels were strongly suppressed in Med, and the changes in which were 0.46 ± 0.06^ΔΔ^ times at 0.083 h, 0.22 ± 0.01^ΔΔ^ times at 12 h, and 0.30 ± 0.01^ΔΔ^ times at 120 h times (Fig. 1b). The same as GAD_67_, GABA-T gene in Med and the changes of which were 0.57 ± 0.02^ΔΔ^ times at 0.083 h, 0.37 ± 0.03^ΔΔ^ times at 12 h, and 0.55 ± 0.06^ΔΔ^ times at 120 h (Fig. 1c). However, GABA-T levels were significantly upregulated in Tel and Cer at 120 h, and their volumes were 1.21 ± 0.20** and 1.55 ± 0.14** (*P* < 0.01).

### Analysis of mRNA expressions in peripheral tissues at the SD of DIF

**Fig. 2:**
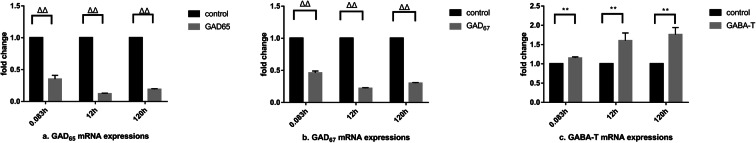
Effects of GAD and GABA-T mRNA expressions in the liver at the SD of DIF

**Fig. 3:**
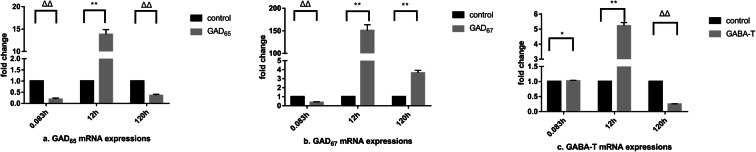
Effects of GAD and GABA-T mRNA expressions in the kidney at the SD of DIF

**Fig. 4:**
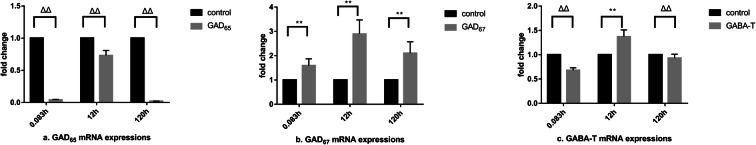
Effects of GAD and GABA-T mRNA expressions in the muscle at the SD of DIF

GAD expressions in the peripheral tissues were extremely significant inhibited by DIF (*P* < 0.01). As shown in Fig. 2 a and b and, GAD_65_ and GAD_67_ genes were suppressed to minimums for 0.12 ± 0.01^ΔΔ^ and 0.22 ± 0.01^ΔΔ^ at 12 h, while GABA-T genes were significantly upregulated in the liver. The maximum changes of GABA-T were 1.76 ± 0.18** times at 120 h in the liver (Fig. 2c). GAD and GABA-T levels appeared consistent trends as first decreasing, then increasing, and decreasing in the kidney. Among them, GAD_65_ levels were 0.19 ± 0.05^ΔΔ^ at 0.083 h, 13.81 ± 1.06** at 12 h, and 0.36 ± 0.05^ΔΔ^ at 120 h (Fig. 3a), while GAD_67_ levels were 0.37 ± 0.08^ΔΔ^ at 0.083 h, 150.67 ± 12.85** at 12 h, and 3.64 ± 0.28* at 120 h (Fig. 3b). Moreover, GABA-T gene were 1.02 ± 0.02* at 0.083 h, 5.22 ± 0.22** at 12 h, and 0.25 ± 0.01^ΔΔ^ at 120 h (Fig. 3c). GAD_65_ level was greatly inhibited in the muscle, and its minimum value was 0.04 ± 0.008^ΔΔ^ at 0.083 h (Fig. 4a), while GAD_67_ level was extremely significant increased with a maximum change of 2.89 ± 0.58** times at 12 h (Fig. 4b). GABA-T levels were 0.68 ± 0.05^ΔΔ^ at 0.083 h, 1.37 ± 0.14** at 12 h, and 0.93 ± 0.08^ΔΔ^ at 120 h (Fig. 4c), which also showed a same trend as “decreasing—increasing—decreasing.”

### Analysis of mRNA expressions at LD_50_ of DIF

The GAD_65_, GAD_67_, and GABA-T levels were extremely increased both in the brain and peripheral tissues of the crucian carp at LD_50_ of DIF (*P* < 0.01, Tables 2 and 3). The volumes of extremely stimulated GAD_65_, GAD_67_, and GABA-T levels were 2.25 ± 0.16** in Cer, 2.41 ± 0.09** in Tel, and 1.97 ± 0.23** in Med (Table 2). As in the kidney, GAD_65_ and GAD_67_ levels were significantly increased by 3.81 ± 0.19** and 8.46 ± 1.43**. A phenomenon in the muscle was that GAD_65_ level was suppressed by 0.32 ± 0.03^ΔΔ^ times at 0.083 h, while GAD_67_ and GABA-T levels were raised to 14.19 ± 1.25** times and 1.12 ± 0.18** times (Table 2).

**Table 2:** GAD_65_, GAD_67_, and GABA-T mRNA expressions at LD_50_ of DIF

Tissues	GAD_65_	GAD_67_	GABA-T
Tel	1.02 ± 0.14	2.41 ± 0.09**	1.04 ± 0.16**
Mer	1.08 ± 0.8**	1.38 ± 0.23**	1.03 ± 0.11**
Cer	2.25 ± 0.16**	1.79 ± 0.06**	1.12 ± 0.18**
Med	1.41 ± 0.07**	1.57 ± 0.11**	1.97 ± 0.23**
Liver	1.41 ± 0.07**	1.57 ± 0.21**	1.04 ± 0.16**
Kidney	3.81 ± 0.19**	8.46 ± 1.43**	1.03 ± 0.11**
Muscle	0.32 ± 0.03^ΔΔ^	14.19 ± 1.25**	1.12 ± 0.18**

**Table 3:** GAD_65_, GAD_67_ and GABA-T mRNA expressions at LC_50_ of AVM

Tissues	GAD_65_	GAD_67_	GABA-T
Tel	0.17 ± 0.03^ΔΔ^	0.32 ± 0.05^ΔΔ^	0.83 ± 0.02^ΔΔ^
Mer	0.12 ± 0.01^ΔΔ^	0.23 ± 0.02^ΔΔ^	0.66 ± 0.05^ΔΔ^
Cer	0.41 ± 0.02^ΔΔ^	0.61 ± 0.09^ΔΔ^	0.68 ± 0.05^ΔΔ^
Med	0.23 ± 0.03^ΔΔ^	0.19 ± 0.01^ΔΔ^	0.64 ± 0.07^ΔΔ^
Liver	0.27 ± 0.03^ΔΔ^	0.23 ± 0.02^ΔΔ^	0.53 ± 0.06^ΔΔ^
Kidney	0.36 ± 0.06^ΔΔ^	0.35 ± 0.04^ΔΔ^	0.91 ± 0.09^ΔΔ^
Muscle(× 10^−1^)	0.029 ± 0.002^ΔΔ^	0.62 ± 0.02^ΔΔ^	0.53 ± 0.05^ΔΔ^

### Analysis of mRNA expressions in the brain at the SC of AVM

**Fig. 5:**
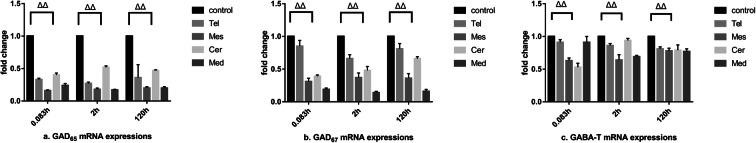
Effects of GAD and GABA-T mRNA expressions in the brain at the SC of AVM

Note: 0.083 h, 2 h, and 120 h were the time points of AVM at the SC (0.0039 mg/L) (Ruan et al. 2013).

Treated by AVM, the levels of GAD_65_, GAD_67_, and GABA-T were extremely inhibited in the brain (*P* < 0.01, Fig. 5). The volumes and the time-points which GAD_65_ expressions remarkably inhibited were 0.16 ± 0.01^ΔΔ^ at 0.083 h in Mer, 0.17 ± 0.01^ΔΔ^ at 2 h in Med, and 0.20 ± 0.02^ΔΔ^ at 120 h in Mes, respectively (Fig. 5a). There were significantly downregulated GAD_67_ gene expressions in Med, which of the gene levels were 0.19 ± 0.02^ΔΔ^ at 0.083 h, 0.14 ± 0.02^ΔΔ^ at 2 h, and 0.16 ± 0.03^ΔΔ^ at 120 h (Fig. 5b). In contrast, the GABA-T level was less inhibited, with which was minimum by 0.53 ± 0.06 times at 0.083 h in Cer (Fig. 5c).

### Analysis of mRNA expressions in peripheral tissues at the SC of AVM

**Fig. 6:**
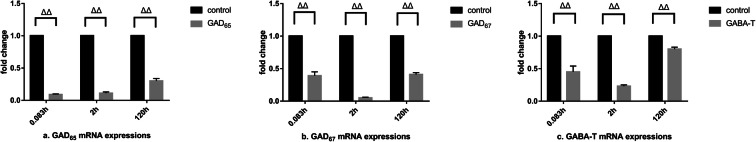
Effects of GAD and GABA-T mRNA expressions in the liver at the SC of AVM

**Fig. 7:**
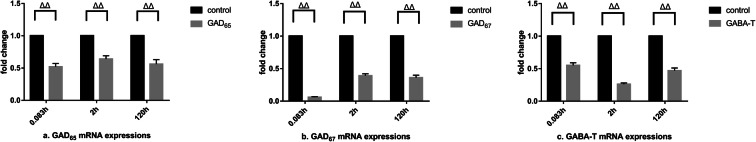
Effects of GAD and GABA-T mRNA expressions in the kidney at the SC of AVM

**Fig. 8:**
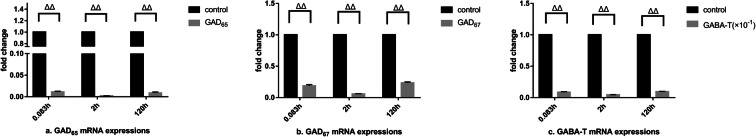
Effects of GAD and GABA-T mRNA expressions in the muscle at the SC of AVM

According to Fig. 6, the GAD and GABA-T levels in the liver were both strongly inhibited at the SC of AVM (*P* < 0.01, Fig. 6). GAD_65_ and GAD_67_ levels were remarkably suppressed to 0.09 ± 0.01^ΔΔ^ at 0.083 h (Fig. 6a) and 0.05 ± 0.01^ΔΔ^ at 2 h (Fig. 6b). GABA-T genes were inhibited significantly, with minimum of which was 0.23 ± 0.02^ΔΔ^ at 2 h in the liver (Fig. 6c), and expressions of GABA-T showed as a “high-low-high” trend. The GAD and GABA-T levels were also significantly downregulated in the kidney. In general, the volumes and the time-points which GAD_65_, GAD_67_, and GABA-T levels remarkably inhibited were 0.52 ± 0.05^ΔΔ^ at 0.083 h (Fig. 7a), 0.06 ± 0.01^ΔΔ^ at 0.083 h (Fig. 7b), and 0.26 ± 0.02^ΔΔ^ at 2 h (Fig. 7c), respectively. Similarly, GAD and GABA-T levels in the muscle were also inhibited strongly. The largest variation range of GAD_65_, GAD_67_, and GABA-T were 0.20 ± 0.02^ΔΔ^ × 10^−2^ (Fig. 8a), 0.57 ± 0.06^ΔΔ^ × 10^−1^ (Fig. 8b), and 0.44 ± 0.02^ΔΔ^ × 10^−1^ times greater (Fig. 8c) at 2 h, respectively.

### Analysis of mRNA expressions at the LC_50_ of AVM

GAD_65_, GAD_67_, and GABA-T levels both in the brain and peripheral tissues were significantly downregulated (*P* < 0.01) at 96 h LC_50_ (0.039 mg/L). GAD_65_ level was inhibited significantly to 0.12 ± 0.01^ΔΔ^ in Mer. Furthermore, GAD_67_ and GABA-T levels with minimum values were 0.19 ± 0.01^ΔΔ^ and 0.64 ± 0.07^ΔΔ^ in Med (Table 3). Compared to the liver, GAD_65_, GAD_67_, and GABA-T levels in the kidney were less inhibited. And the mRNA expressions of GAD_65_, GAD_67_, and GABA-T were 0.36 ± 0.06^ΔΔ^, 0.35 ± 0.04^ΔΔ^, and 0.91 ± 0.09^ΔΔ^, respectively. GAD and GABA-T levels in the muscle were inhibited strongly, where the relative expressions of GAD_65_, GAD_67_, and GABA-T were 0.0029 ± 0.0002^ΔΔ^, 0.062 ± 0.002^ΔΔ^, and 0.053 ± 0.005^ΔΔ^, respectively (Table 3).

## Discussions

### Effects of DIF on the mRNA expressions of GAD and GABA-T in crucian carp

It is generally believed that FQs antagonizes inhibitory neurotransmitter GABA, thereby increasing nerve excitability, leading to convulsions, epilepsy, and other adverse reactions (Motomura et al. 1991; Matsuo et al. 1998). GABA mediates the release of inhibitory synapses of neurons, which can reduce the hyperexcitability of neurons. Previous study found that crucian carp suffered from impatience and restlessness, body type tremors when treated with DIF (Ruan et al. 2014a). Meanwhile, it was also found in this paper that GAD_65_, GAD_67_, and GABA-T levels were significantly downregulated at 0.083 and 12 h in the brain after administrated with DIF at its SD (20 mg/kg b. W.), while GAD_65_, GAD_67_, and GABA-T levels were significantly upregulated at LD_50_ (2840 mg/kg b. W.) of DIF (*P* < 0.01, Fig. 1 and Table 2). However, GABA-T levels were significantly upregulated at 120 h in Tel and Cer after treated with DIF at its SD, which seemed to suggest that DIF could stimulate the overexpression of GABA-T to consume the GABA flux in the nerve center as an antagonistic inhibitor of GABA and enhance the convulsion effect of crucian carp. Moreover, the decreased GABA levels were also reported in the albino rat brain after intraperitoneal injection of FQS (Arafa et al. 2015). Similarly, GABA was continuously inhibited, resulting in epilepsy of old people after treated with FQS (Isaacson et al. 1993). These pieces of evidence suggested that the systemic neurotoxicity of crucian carp may be related to the upregulation of mRNA levels of GAD and GABA-T in various tissues treated with the lethal dose of DIF.

After the treatments at SD or LD_50_ of DIF, GABA-T level was significantly increased (*P* < 0.01) in the liver (Fig. 2 and Table 2). While GAD levels were of different expressions at the two doses, which may be lead to the accumulation of GABA in the liver for the reason of DIF. In addition, GAD and GABA-T levels showed time-concentration effect after administration with DIF at its SD or LD_50_ in the kidney (Fig. 4). This may suggested that low dose of DIF would inhibit GABA pathway, while high dose (or high residual) has opposite performance, the same as the effect of DIF on the central nervous system. This phenomenon may be a protective mechanism of stress resistance in crucian carp.

### Effects of AVM on the mRNA expressions of GAD and GABA-T in crucian carp

GABA levels in organisms were determined by the dynamic balance between synthesis and catabolism and regulated by the level of GAD, precursor availability, and possibly GABA degradation (de Graaf et al. 2006). It has been reported that the neurotoxicity of AVM to organisms was due to its ability to trigger the opening of Cl^−^ channels (Lasota and Dybas 1991). This process was irreversible and only occurred in invertebrates (Cornejo et al. 2014). In addition, the neurotoxicity of AVM was also reflected in the destruction of a large number of nerve cells (Shu et al. 2010). After been exposed to AVM at the SC (0.0039 mg/L) and LC_50_ (0.039 mg/L), the mRNA expressions of GAD and GABA-T both in the brain and peripheral tissues of crucian carp were inhibited significantly in this paper (*P* < 0.01, Figs. 4, 5, 6, 7, 8 and Table 3). This result indicated that the central nervous system was influenced by AVM in crucian carp. It was found that the mRNA levels of GAD_65_, GAD_67_, and GABA-T in the goldfish’s brain were downregulated after treatment with GABA receptor agonists (Martyniuk et al. 2007). Similar results were found in this paper.

Interestingly, GABA-T was inhibited to a much lower degree than that of GAD’s in the brain at LC_50_ of AVM. Based on previous research, the GAD level was much higher than that of GABA-T in the brain, which seemed to indicate that AVM would causes different expression of GAD and GABA-T (Ruan et al. 2014b). But on the contrary, GAD and GABA-T levels were extremely inhibited in the muscle after treated with AVM at the SC or at the LC_50_ (Fig. 8 and Table 3), which suggested that neuromuscular synthesis and metabolic rate of GABA were stagnant. This may lead to an imbalance in muscular nerve regulation, such as pathological convulsion of muscle. Similar findings were also found that the status of crucian carp was in physical imbalance and has a slower respiration rate after AVM treatments (Wang and Lu 2010). After been exposed to AVM, it was discovered that Japanese quails has a decrease of response to its natural enemies (de Faria et al. 2018b). Other paper found that AVM would cause twitching and keep exciting in bees; this may relate to the fact that AVM inhibits the expressions of GAD and GABA-T in the cerebellum (Zhao et al. 2014). Besides, deltamethrin and *β*-cypermethrin could downregulate GABA-T level in the cerebral cortex of rats, which resulting in an increase in GABA level (Ji et al. 2003; Han et al. 2014). All the evidences mentioned above suggested that AVM would break through the blood-brain barrier, which lead to the increase of GABA through affecting the mRNA expressions of GAD_65_, GAD_67_, and GABA-T in crucian carp’s nervous system.

## Conclusion

The expressions of GAD_65_, GAD_67_, and GABA-T were all significantly downregulated at the SD of DIF except for the upregulated expression of GABA-T in the kidney and muscle tissues at 120 h, while the expressions of the three genes were significantly upregulated at the LD_50_ of DIF. In addition, the expressions of GAD_65_, GAD_67_, and GABA-T in various tissues of crucian carp were significantly downregulated both at the SC and LC_50_ of AVM.
